# Novel nano bearings constructed by physical adsorption

**DOI:** 10.1038/srep14539

**Published:** 2015-09-28

**Authors:** Yongbin Zhang

**Affiliations:** 1 College of Mechanical Engineering, Changzhou University, Changzhou, 213016, Jiangsu Province, China

## Abstract

The paper proposes a novel nano bearing formed by the physical adsorption of the confined fluid to the solid wall. The bearing is formed between two parallel smooth solid plane walls sliding against one another, where conventional hydrodynamic lubrication theory predicted no lubricating effect. In this bearing, the stationary solid wall is divided into two subzones which respectively have different interaction strengths with the lubricating fluid. It leads to different physical adsorption and slip properties of the lubricating fluid at the stationary solid wall respectively in these two subzones. It was found that a significant load-carrying capacity of the bearing can be generated for low lubricating film thicknesses, because of the strong physical adsorption and non-continuum effects of the lubricating film.

In micro/nano devices, the coupled solid surfaces are often parallel to and slide against one another^1^. The lubrication between these surfaces is challenging since it is very important for the performance of the formed contact including the reduction of the adhesive forces between the two surfaces, however conventional hydrodynamic lubrication theory said that no lubrication can be generated there, because of the mass flow rate of the Couette flow of the lubricant entrained into the contact equal to that entrained out of the contact and then the always satisfied flow continuity condition in this contact with vanishing pressure gradients^2^,^3^.

The reasons for the failure of conventional hydrodynamic lubrication theory to predict the performance of a lubricated micro/nano contact are that such a theory only considered homogeneous contact surface properties, neglected the factors of the physical adsorption and the slippage of the lubricating film at the contact surface, and was based on the assumption of a continuum lubricant^4^. When the separation between two coupled solid surfaces is on the nanometer scale, conventional hydrodynamic lubrication theory will surely fail because of the above mentioned unrealistic assumptions taken in this theory. Experiments and molecular dynamics simulations (MDS) have shown that in a nanometer-scale surface separation the confined fluid may severely slip at the solid surface and the contact-fluid interaction strength has a very significant influence on this interfacial slippage^5^,^6^,^7^,^8^,^9^,^10^. Also, in such a separation the physical adsorption of the confined fluid to the solid surface, which appears as the ordering of the confined fluid to the solid surface, may play an important effect on the momentum transfer within the fluid^11^,^12^,^13^,^14^,^15^,^16^. The boundary film interfacial slippage in a lubricated micro/nano contact is the result of the boundary film interfacial shear stress exceeding the boundary film-contact interfacial shear strength^17^. Lower the boundary film-contact interfacial shear strength, higher the boundary film interfacial slipping velocity^17^. The relative slip amount of the boundary film (defined as *γ*_*s*_ in the latter) ranges between −1 and 1. A higher magnitude of *γ*_*s*_ indicates a more severe interfacial slippage, and a magnitude of *γ*_*s*_ equal to unity shows the most severe interfacial slippage, which makes the boundary film on the contact surfaces halted in motion or the greatest increases the moving velocity of the boundary film on the contact surface. The boundary film interfacial slippage can thus reduce or increase the mass flow rate of the lubricating film through the contact depending on the operating condition and where the interfacial slippage occurs. It may thus be harmful or beneficial for the performance of a lubricated micro/nano contact. A strong, medium-level or weak adsorption of the confined fluid to the solid wall in a nano channel may be unavoidably present depending on the interaction strength between the confined fluid and the wall^11^,^12^,^13^,^14^,^15^. Stronger the fluid-wall interaction, stronger the physical adsorption of the fluid to the wall, but weaker the fluid interfacial slippage, or vice versa^11^,^12^,^13^,^14^,^15^.

On the other hand, molecular dynamics simulations showed that the fluid confined in a nano channel actually exhibits non-continuum properties showing varying local densities and viscosities across the channel height^11^,^12^,^13^,^14^,^15^. The flowing laws of the non-continuum fluid in both the Couette and Poiseuille flows are largely different from those of the continuum fluid^11^,^12^,^13^,^14^,^15^. The author showed that the fluid non-continuum effect i.e. the fluid discontinuity and inhomogeneity effects across the fluid film thickness should be responsible for the deviation of a nano channel flow from conventional hydrodynamic lubrication theory description^18^,^19^.

The lubrication in a micro/nano contact may actually be the physical adsorbed layer boundary lubrication, which heavily depends on the interaction between the adsorbed layer and the contact surface. In this regime, the lubrication does not follow conventional hydrodynamic lubrication theory, and a new theory taking into account the physical adsorption, interfacial slippage and non-continuum effects of the boundary film may need to be developed^20^. A physical adsorbed layer boundary lubrication may also be difficult to generate between two parallel smooth surfaces with homogeneous surface properties according to the corresponding developed lubrication theory^21^, because of the vanishing non-continuum and physical adsorption effects of the boundary film. In this case, a physical adsorbed boundary layer is easily squeezed out of the contact under a load and the lubricating effect is actually very difficult to generate.

Continuum hydrodynamic lubrication theory has shown that hydrodynamic lubrication can be generated between two parallel smooth solid surfaces sliding against one another, by artificially designing the contact-fluid interfacial slippage at the specific surfaces^22^. Such a technology may also be applicable for generating a boundary lubrication effect between two sliding parallel smooth solid surfaces including for designing a nano bearing formed by two parallel smooth solid walls^21^. Except the interfacial slippage technology^21^, no other technologies have however been seen for developing the nano slider bearing formed between two parallel smooth solid walls.

The boundary lubrication in a nano contact was ever studied by atomistic simulations (including molecular dynamics simulation and Monte Carlo simulation), which however were usually quite time taking and computer storage consuming and were often only applicable for very small contact areas^23^,^24^,^25^. Such approaches are obviously insufficient for a realistic contact with a much bigger contact size. The ab initio calculation has been found to be not applicable for a micro contact with big contact width and/or contact length. For overcoming the massive time taking and the huge computer storage consumption in such a calculation, a lot of computational methods were ever developed, however still far away from being applicable to a realistic contact^26^.

A lot of continuum or quasi-continuum models have also been proposed for simulating the flow of a fluid confined in a nano channel. Hansen *et al.*^27^ ever derived the velocity profiles across the channel height of both the Couette and Poiseuille flows by using the Navier-Stokes equation, considering both the interfacial slippage on the solid wall and the intermolecular forces in the system. Although both the slip length and the film velocity profile in the Poiseuille flow calculated from their model matched the MDS results, their model was based on the continuum fluid assumption, lacking in incorporating the discontinuity and inhomogeneity of the confined fluid across the channel height. Bhatia *et al.*^28^ reviewed the Knudsen model, the dusty gas model, the interfacial friction-based model, the Maxwell-Stefan approach, the oscillator model and the distributed friction approach for theoretically studying the molecular transport in nanopores, and pointed out the characteristic and shortcomings of these models respectively. Some of them omitted the confined luid-wall interaction, while the others only implicitly considered the inhomogeneity of the confined fluid although they incorporated the fluid-wall interaction. Giannakopoulos *et al.*^29^ studied by MDS the size effects of the diffusion coefficient, shear viscosity and thermal conductivity of a simple fluid flowing in a nano channel at a constant temperature. They related these fluid properties to their bulk values respectively by some formulating equations. They showed that the shear viscosity of the confined fluid was increased with the channel height reduction, while the other two fluid properties otherwise. Their study made a connection between the MDS results and the empirical equation formulations of the physical properties of a confined fluid, the latter being important for an efficient modeling of an engineering contact. They later developed a quasi-continuum model for the self-diffusion of a fluid flowing in a nano channel to capture the MDS calculated fluid ordering across the channel height^30^. Their model was an effort to find an efficient approach for simulating a multiscale flow covering from nano to macro.

The interesting phenomena concerning the molecular spin, rotational viscosity and angular momentum of a confined fluid in a nano channel were also studied by MDS. Moore *et al.*^31^ showed that a chlorine fluid or a fluid composed of small linear molecules possess a rotational viscosity. Hansen *et al.*^32^ showed that in a non-steady flow in a nano channel with extremely high oscillating frequencies, within the confined fluid the angular momentum should be coupled with the translational momentum by considering the fluid rotational viscosity, and the molecular spin effect of the confined fluid was not negligible. However, they showed that for a steady flow or a non-steady flow with a low Reynolds number these two momentums can be decoupled from one another and the molecular spin effect of the confined fluid is negligible.

The flow factor approach model has been proposed for studying the physical adsorbed layer boundary lubrication^33^. The nature of this model was recently uncovered to well agree with the MDS results in the calculated flowing velocities of the confined fluid both in the Couette and Poiseuille flows^18^,^19^. The flow equation of the confined fluid in a nano channel was then derived from this model^34^. The model neglects the molecular spin effect of the confined fluid and is suitable for low Reynolds numbers. It is actually an equivalent continuum model incorporating both the dynamic and non-continuum effects of the confined fluid, by considering the fluid discontinuity and inhomogeneity across the channel height. The advantages of this model are that it not only captures the MDS calculated characteristic of the flow of the confined fluid but also is efficiently applicable for a realistic contact with a big contact size. Based on this model, the analytical results for a nano step bearing were obtained^21^,^34^.

The flow factor approach model as well as the molecular dynamics simulation results showed that the average velocity across the channel height of a confined fluid in a nano channel in the Couette flow is indeed equal to that calculated from conventional hydrodynamic lubrication theory, while the magnitude of the flowing velocity of this confined fluid in the Poiseuille flow is reduced because of the non-continuum effect i.e. the discontinuity and inhomogeneity effects of the confined fluid across the channel height^18^,^19^. These results may give important indications for the mechanism of the load-carrying capacity generation in a nano bearing. That is, even with two parallel smooth solid walls, if the interaction between the fluid and the solid wall in the inlet zone is stronger than that in the outlet zone, significant pressures and then the load-carrying capacity would be generated in a nano slider bearing, because of the mass flow rate in the Couette flow into the bearing greater than that out of the bearing owing to the stronger adsorption to the wall of the fluid and then the higher average fluid density across the fluid film thickness in the inlet zone than in the outlet zone. The non-continuum effect of the fluid in a nano bearing reduces the magnitudes of the mass flow rate of the Poiseuille flow both in the bearing inlet and outlet zones, it then increases the magnitude of the total mass flow rate into the bearing but reduces the magnitude of the mass flow rate out of the bearing. For maintaining the flow continuity, higher pressures are required to be generated respectively in the bearing inlet and outlet zones to produce the corresponding Poiseuille flows in these two subzones because of the fluid non-continuum effect. The fluid non-continuum effect would thus significantly increase the load-carrying capacity of a nano bearing. On the other hand, the slippage of the confined fluid at the wall surface in a nano bearing would also significantly influence the carried load of the bearing.

According to the underlying mechanisms of the load-carrying capacity generation in a nano bearing, the present paper attempts to study the performance of a novel nano slider bearing formed between two parallel smooth solid plane walls which is dependent on the physical adsorption, by using the flow factor approach model. Different from the previously studied bearing taking the homogeneous surface properties shown in ref. 34, the present bearing takes inhomogeneous surface properties on the stationary solid wall for generating the bearing load-carrying capacity, i.e. in the present bearing the interaction between the lubricating film and the stationary solid wall in the bearing inlet zone is stronger than that in the bearing outlet zone. This purpose could be realized by taking different materials of the stationary solid wall or covering different coatings on the stationary solid wall respectively in the bearing inlet and outlet zones. The present bearing is also different from the studied bearing in ref. 21, which depended on the artificially introduced interfacial slippage in the bearing inlet zone for improving the bearing performance, in the condition that the interaction between the stationary solid wall and the lubricating film in the bearing inlet zone was much weaker than that in the bearing outlet zone. The surface properties of the stationary solid wall and the physical adsorption properties of the lubricating film in the present bearing are thus largely different from those in the studied bearing in ref. 21.

Based on the flow factor approach model, the present paper presents an analysis for the load-carrying capacity of the studied bearing. The condition for the formation of the bearing was obtained. The calculation results showed that significant pressures and then pronounced load-carrying capacities can be generated in the studied bearing for low lubricating film thicknesses, because of the strong physical adsorption and non-continuum effects of the lubricating film. The optimum condition for the maximum load-carrying capacity of the bearing was also obtained.

## Studied bearing

**Figure 1:**
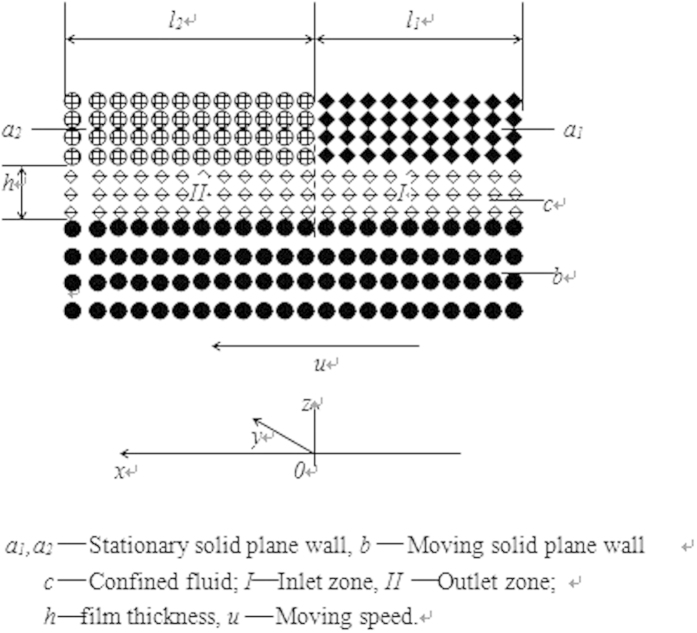
The proposed nano bearing dependent on the physical adsorption of the confined fluid to the solid walls. Description: The material or surface material of the “*a_1_*” subzone are different from those of the “*a_2_*” subzone, and the interaction between the fluid and the wall in the “*a_1_*” subzone is significantly stronger than that between the fluid and the wall in the “*a_2_*” subzone.

Figure 1 shows the studied nano bearing formed by two parallel smooth solid plane walls sliding against one another. The whole moving solid wall is made of identical material, to which the adsorption of the fluid may be strong, medium-level or weak. The confined fluid may slip or not slip at this wall surface depending on its adsorption to this wall surface. The stationary solid wall is divided into two subzones i.e. the “a_1_” and “a_2_” subzones which are respectively made of different materials or covered with different coatings. The adsorption of the fluid to the wall surface in the “*a*_*1*_” subzone is significantly stronger than that to the wall surface in the “*a*_*2*_” subzone. The confined fluid may slip or not slip at the wall surfaces in these two subzones depending on its adsorptions to these wall surfaces.

### Analysis

The whole lubricated area of the studied bearing is divided into the “I” and “II” subzones, which respectively denote the bearing inlet and outlet zones. The Cartesian coordinate system is shown in Fig. 1. In the analysis, it was assumed that the pressure within the confined fluid is constant across the film thickness. This is allowable according to the molecular dynamics simulation results^35^. The assumption of the constant pressure across the film thickness was also taken for the film thickness on the micrometer scale as made in conventional hydrodynamic lubrication theory^3^. The present model is thus not limited to the nanometer scale film thickness, but can actually be extended to the micrometer scale film thickness i.e. the case of a continuum lubricating film as shown by Eqs. (1) and (2) in the latter.

In the “*y*” coordinate direction, the confined fluid was assumed not to flow, i.e. no side leakage was assumed in the bearing. Such an assumption was also often taken in the analysis of a conventional hydrodynamic bearing^3^. It is allowable when the size of the contact in the “*y*” coordinate direction is much bigger than that in the “*x*” coordinate direction so that the fluid side flow is negligible compared to the fluid total flow into the contact. In this case, the assumption can give a good prediction for the load-carrying capacity of the bearing. For the contact size in the “*y*” coordinate direction comparable to that in the “*x*” coordinate direction, the side leakage effect may need to be considered, and a correction factor can be introduced to modify the carried load of the bearing calculated based on the no slide leakage assumption to account for the side leakage effect as ever done in the analysis of the conventional hydrodynamic bearing^3^. The present work is fundamental, and the correction factor for the carried load of the bearing accounting for the side leakage effect can be studied in the subsequent research.

In the present analysis, the frictional heating in the bearing was also neglected. This may be allowable when the sliding speed is low. However, for a high sliding speed, this assumption may be not allowable, and a thermal analysis for the bearing may be required. The present work can give a fundamental result for the studied bearing, and regarding the friction heating effect a correction factor can also be introduced to modify the present obtained results, as ever done in the conventional hydrodynamic bearing analysis^3^.

The coupled wall surfaces in the present bearing were treated as ideally smooth and rigid. These treatments can simplify the problem. For low lubricating film pressures and the protrusion on the wall surface much lower than the lubricating film thickness, these treatments are allowable. However, when the film pressure is so high that the caused wall surface elastic deformation is comparable to the film thickness, the influence of the wall surface elastic deformation on the bearing performance should be considered. On the other hand, when the wall surface protrusion is comparable to the film thickness, the wall surface roughness effect should also be considered. These could be put as interesting topics in the future research. Furthermore, since the present paper addresses the physical adsorption effect on the load-carrying capacity of the bearing, here the wedge effect between the coupled walls is neglected, i.e. the two walls of the bearing are parallel to one another. Nevertheless, the wedge effect between the walls is also important for the carried load of the bearing as has been known from conventional hydrodynamic lubrication theory^3^. The research in this regard will be carried out very soon.

Finally, since the present paper thrusts efforts into the study on the load-carrying capacity of a nano bearing contributed by physical adsorption, the effect of the surface pressures due to the wall interactions on the carried load of the bearing is neglected, i.e. the carried load of the bearing calculated here is contributed by the lubricating film. Nevertheless, the present calculation is usually the main portion of the load-carrying capacity of the bearing and sufficient, as the surface pressure effect is usually weak and negligible when the lubricating film thickness is a bit higher than 1 nm^36^,^37^, and also the contribution of the surface pressures is normally far smaller than the contribution of the lubricating film.

Based on the above mentioned assumptions, according to the flow factor approach model for the flow of the confined fluid in a nano channel^34^, the mass flow rate per unit contact length through the contact of the confined fluid in the “I” subzone is:


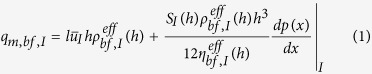


where *h* and *p* are respectively the film thickness and pressure of the confined fluid, *x* is the coordinate in the flow direction of the confined fluid shown in Fig. 1, 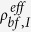 is the average density of the confined fluid across the film thickness in the “I” subzone which is dependent on the film thickness, 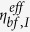 is the effective viscosity of the confined fluid in the “I” subzone which is dependent on the film thickness, 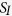 is the parameter depicting the non-continuum effect i.e. the discontinuity and inhomogeneity effects of the confined fluid on the fluid flow in the “I” subzone which is dependent on the film thickness and 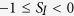, and 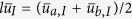. Here, 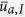 and 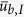 are respectively the velocities of the confined fluid on the stationary and moving solid wall surfaces in the “I” subzone. When 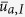 and 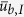 are respectively equal to the moving speeds of the corresponding contact surfaces, no interfacial slippage occurs; Otherwise, interfacial slippage will occur. The first term on the right-hand side of Eq. (1) is the Couette flow and it is exact according to the MDS results^18^,^19^, and the second term on this side is the Poiseuille flow. As Eq. (1) shows, the non-continuum effect of the confined fluid is reflected in the term of the Poiseuille flow. For a vanishing pressure gradient, the Poiseuille flow vanishes and the non-continuum effect of the confined fluid then disappears. On the other hand, for a film thickness greater than the critical film thickness 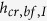, the value of 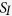 in Eq. (1) is −1 so that Eq. (1) is reduced to the Reynolds equation which is for a continuum fluid lubrication; Otherwise, for a lower film thickness, the magnitude of 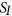 is smaller (less than unity), and the non-continuum effect of the confined fluid is stronger. Stronger the non-continuum effect of the confined fluid, greater the reduction of the magnitude of the Poiseuille flow. The magnitude of *S* is actually the ratio of the Poiseuille flow rate of the non-continuum fluid to that of an equivalent continuum fluid. 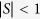 was substantiated by the MDS results^15^,^18^,^19^, which showed that the non-continuum effect (i.e. the discontinuity and inhomogeneity effects across the film thickness) of the confined fluid reduces the magnitude of the flowing velocity of the Poiseuille flow of the confined fluid. By taking the physical properties 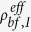, 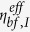 and 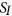 of the confined fluid and the entraining velocity 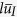 all dependent on the fluid-wall interaction, equation (1) takes into account the interfacial slippage, physical adsorption and non-continuum effects of the confined fluid. When the film thickness *h* is below the critical film thickness 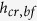, the lubricant film between two solid walls will become non-continuum across the film thickness, conventional (continuum) hydrodynamic lubrication theory will fail, and a corresponding non-continuum lubrication theory such as shown by Eq. (1) should be applied. For a common oil such as paraffinic or synthetic oils, 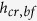 is normally on the scale of 10 nm^20^; While for a simple liquid such as liquid argon and water, 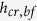 may only be on the scale of 1 nm^11^,^15^.

A conventional (continuum) hydrodynamic lubrication theory was ever used for studying the performance of a molecularly thin film lubricated contact by incorporating the surface pressures because of the wall interactions^38^. Such an approach suffered from the critical shortcomings in modeling the rheology of the confined non-continuum fluid and should normally give the load-carrying capacity of the lubricated contact much lower than that given by a non-continuum lubrication theory such as shown by Eq. (1) because of neglecting the physical adsorption and non-continuum effects of the lubricating film^21^,^34^. However, when the lubricant film is so thick that its thickness is not considerably lower than the critical film thickness 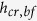, the physical adsorption and non-continuum effects of the confined film may be weak and negligible, in this case such an approach may give the carried load of the lubricated contact close to that given by a non-continuum lubrication theory^21^,^34^.

A similar equation can be implemented for the “II” subzone and it reads:


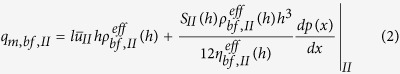


where the meanings of the respective parameters are respectively same with those in Eq. (1) except that the subscript “II” denotes the “II” subzone.

As the flow continuity requires, 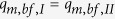; If 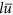, 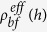, 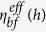 and *S*(*h*) in Eqs. (1) and (2) are known, for a given *h*, the pressure distributions in the “I” and “II” subzones can respectively be solved from these two equations by using the flow continuity condition and the pressure boundary conditions 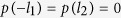. At the same time, the mass flow rates 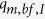 and 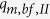 will be solved out.

Because the adsorption of the confined fluid to the wall surface in the “*a*_*1*_” subzone is stronger than that to the wall surface in the “*a*_*2*_” subzone, 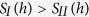, 
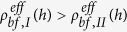
 and 
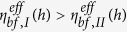
34. In this condition, the non-continuum effect of the confined fluid in the “I” subzone is stronger than that in the “II” subzone.

If 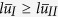, we have 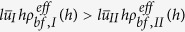. As Eqs. (1) and (2) show, in this condition, the fluid mass flow rate in the Couette flow in the “I” subzone is greater than that in the “II” subzone. Thus in this case, if there were no pressures generated in the contact, the flow continuity would not be maintained in the contact. In such a condition, for maintaining the flow continuity, the pressures in the contact must be generated to produce the corresponding Poiseuille flow to balance the total flow. Obviously, in this condition, the bearing is formed with a certain load-carrying capacity.

**Figure 2:**
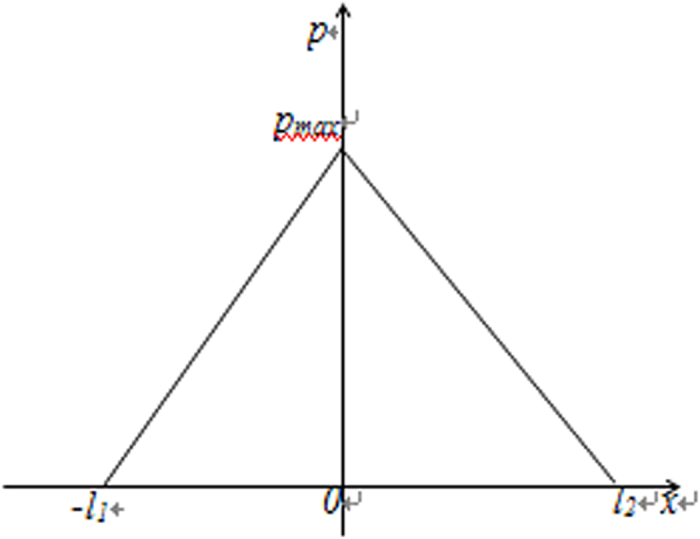
An illustration of the pressure distribution in the present bearing. 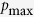
 *is the maximum pressure in the bearing.* Description: The pressures in the “I” and “II” subzones in the bearing are respectively linearly distributed and the maximum pressure occurs on the boundary between these two subzones.

As the flow continuity requires, for a given operating condition, 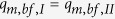
=constant. Thus, according to Eq. (1), if 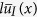 is constant, 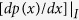 is constant for a given *h*. For the same, if 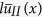 is constant, 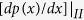 is constant for a given *h*. As Fig. 1 shows, the pressure boundary conditions are: 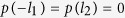. Let 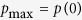, for constant 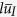 and 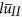, the distribution of the pressure within the confined fluid is shown in Fig. 2. It is shown that in this condition the pressures in the “I” and “II” subzones are respectively linearly distributed and the maximum pressure 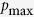 occurs on the boundary between these two subzones.

Let 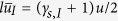 and 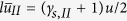. Here, *u* is the speed of the moving solid wall, and 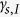 and 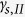 are respectively the relative slip amounts of the confined fluid in the “I” and “II” subzones, which depends on the fluid-wall interfacial shear strengths in these two subzones and the operating condition^21^. Here, for a given operating condition, 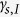 and 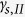 are respectively constant, and their values are given beforehand presumably. 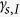 and 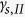 are dimensionless and entirely different from the conventionally defined (dimensional) slip length, which was used to measure the degree of the interfacial slippage^39^. Here, 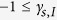, 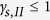, a higher magnitude of 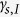 or 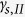 indicates a more severe interfacial slippage in the “I” or “II” subzones, while the magnitudes of 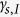 or 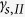 equal to unity indicates the most severe interfacial slippage in the “I” or “II” subzones. Both the signs of 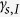 and 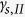 can be positive or negative depending on whether the interfacial slippage increases the Couette flows in the “I” and “II” subzones. For 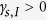, 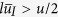 and the interfacial slippage in the “I” subzone makes the Couette flow of the confined fluid greater than that without the interfacial slippage; This results in a more mass flow rate entrained into the contact and consequently higher film pressures and load-carrying capacity of the bearing. Thus, 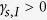 is beneficial for the load-carrying capacity of the bearing, while 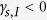 harmful. The influence of 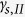 is contrary; 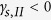 is beneficial for the load-carrying capacity of the bearing, while 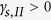 harmful.

For a given operating condition, if the interfacial slippage occurs, 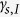 and 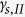 have been found to be respectively constant for the contact formed by two parallel solid plane surfaces^21^,^40^. This leads to constant 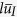 and 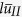 in such a contact for a given operating condition. Moreover, when the sliding speed *u* is high enough, 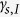 and 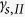 can respectively be taken as constants independent on *u*^40^.

### Pressure and carried load

For constant 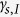 and 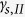, according to 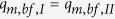 and the pressure distribution shown in Fig. 2, it is obtained that:


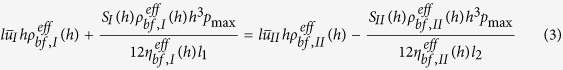


It is solved from Eq. (3) that:


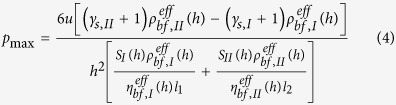


The load per unit contact length carried by the bearing is:


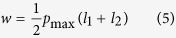


### Normalization

Define the following dimensionless parameters:


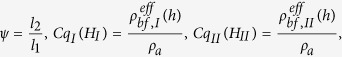



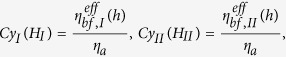



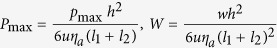


Here, 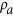 and 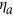 are respectively the density and viscosity of the fluid at ambient condition when the fluid is continuum; 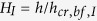, 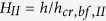, and 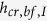 and 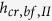 are respectively the critical film thicknesses of the confined fluid in the “I” and “II” subzones for the confined fluid in these subzones to become continuum across the film thickness.

The dimensionless maximum pressure is:


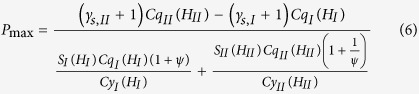


The dimensionless load carried by the bearing is:


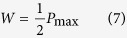


### Mechanisms for the load-carrying capacity of the bearing

According to Eq. (6), the mechanisms of the load-carrying capacity of the present bearing can be drawn as follows:

1. For no interfacial slippage occurrence i.e. 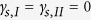, 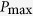 is determined by the term 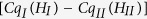. In this case, the load-carrying capacity of the bearing is generated because of the different physical adsorptions of the confined fluid to the stationary wall surfaces respectively in the “I” and “II” subzones.
2. If 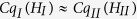, 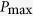 is determined by the term 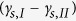 which should be positive. In this case, the load-carrying capacity of the bearing is generated because of the different interfacial slippage respectively in the “I” and “II” subzones. For example, such a load-carrying capacity can be attained by respectively designing the low fluid-wall interfacial shear strength at the wall surface of the “a_1_” subzone and the relatively high fluid-wall interfacial shear strength at the wall surface of the “a_2_” subzone so that the fluid slippage respectively occurs at the “a_1_” wall surface in the “I” subzone and at the moving wall surface in the “II” subzone. In this case, 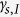 > 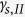
21. On the other hand, it is obtained that for the interfacial slippage in the “I” subzone helpful for the generation of the load-carrying capacity of the bearing, 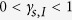, otherwise 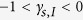; While, for the interfacial slippage in the “II” subzone helpful for the generation of the load-carrying capacity of the bearing, 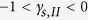, otherwise 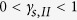.
3. Actually, the load-carrying mechanism of the bearing may be the combined effects of the physical adsorption, non-continuum and interfacial slippage of the confined fluid. The condition for the formation of the bearing is: 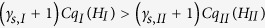. If 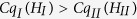, for 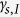 = 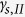, the condition for the formation of the bearing is always satisfied and the load-carrying capacity of the bearing will be generated.
4. Because 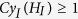, 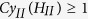, 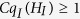, 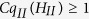, 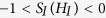 and 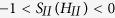 21,

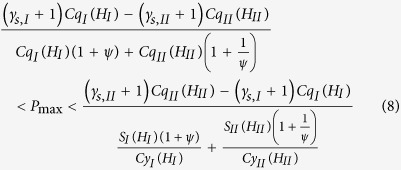


(5) Optimum geometry of the bearing:

The optimum value of *ψ* for the maximum load-carrying capacity of the bearing is:


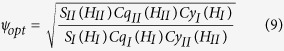


In this optimum condition, the dimensionless load carried by the bearing is:


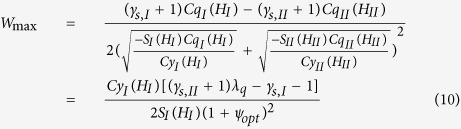


where 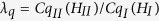.

From Eq. (10), it can be found that lower magnitudes of 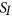 and 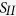 but higher magnitudes of 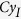 and 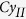 gives higher load-carrying capacities of the bearing. For example, for a given film thickness *h*, increasing both the interaction strengths between the confined fluid and the “a_1_” and “a_2_” walls yields lower magnitudes of 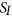 and 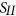 but higher magnitudes of 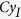 and 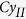
11,18, and it can thus increase the load-carrying capacity of the bearing.

### Calculation

The parameters 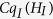 and 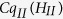 are expressed as the following general form^41^:


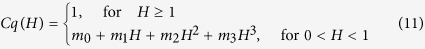


where 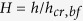, and 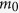, 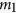, 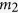 and 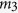 are respectively constants.

The parameters 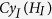 and 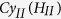 are expressed as the following general form^41^:


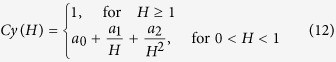


where 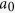, 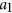 and 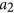 are respectively constants.

The parameters 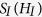 and 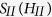 are expressed as the following general form^41^:


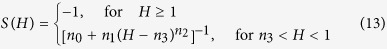


where 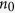, 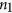, 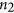 and 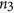 are respectively constants.

Exemplary calculations were made. In these calculations, the interaction between the confined fluid and the wall in the “a_2_” subzone is relatively weak, and the interaction between the confined fluid and the wall in the “a_1_” subzone is respectively taken as medium-level and relatively strong. In the calculations, 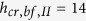 *nm*, 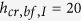 *nm* for the medium-level interaction between the confined fluid and the wall in the “a_1_” subzone, and 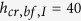 *nm* for the relatively strong interaction between the confined fluid and the wall in the “a_1_” subzone. The values of 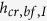 and 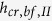 were chosen according to the experimental observations^16^,^20^. The values of the other parameters in the calculation are respectively shown in Tables 1, 2 and 3. The comparisons of the values of 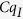, 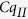, 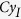, 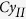, 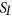 and 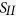 for different film thicknesses based on the input parameter values in Tables 1, 2 and 3 were shown in ref. 41. These dimensionless fluid rheological parameter values well agree with the experimental measurements and molecular dynamics simulation results^11^,^12^,^13^,^14^,^15^,^16^.

## Results and discussion

### Typical results

**Figure 3:**
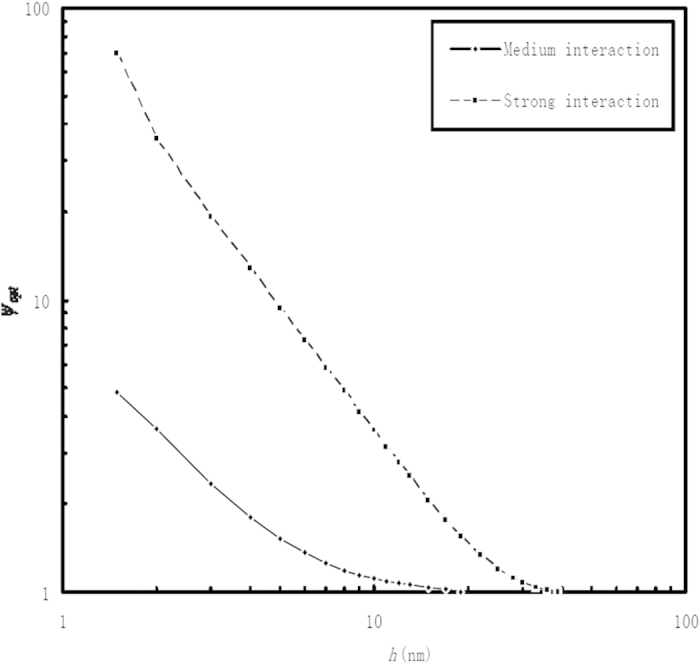
Plots of the value of 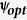 against the film thickness *h*when the interaction between the confined fluid and the wall in the “a_1_ ” subzone is respectively relatively strong and medium-level. 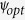
 *is the optimum value of* 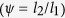 *for the maximum load-carrying capacity of the bearing.* Description: The value of 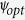 is independent on the interfacial slippage but heavily dependent on the interactions between the confined fluid and the walls in the “a_1_” and “a_2_” subzones.

Figure 3 shows the values of 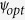 for different film thicknesses when the interaction between the confined fluid and the wall in the “a_1_” subzone is respectively relatively strong and medium-level. The value of 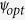 is independent on the interfacial slippage but heavily dependent on the interactions between the confined fluid and the walls in the “a_1_” and “a_2_” subzones. For a given nanometer-scale film thickness *h*, the increase of the adsorption of the confined fluid to the wall in the “a_1_” subzone significantly increases the value of 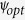; While, the film thickness increase significantly reduces the value of 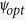.

**Figure 4:**
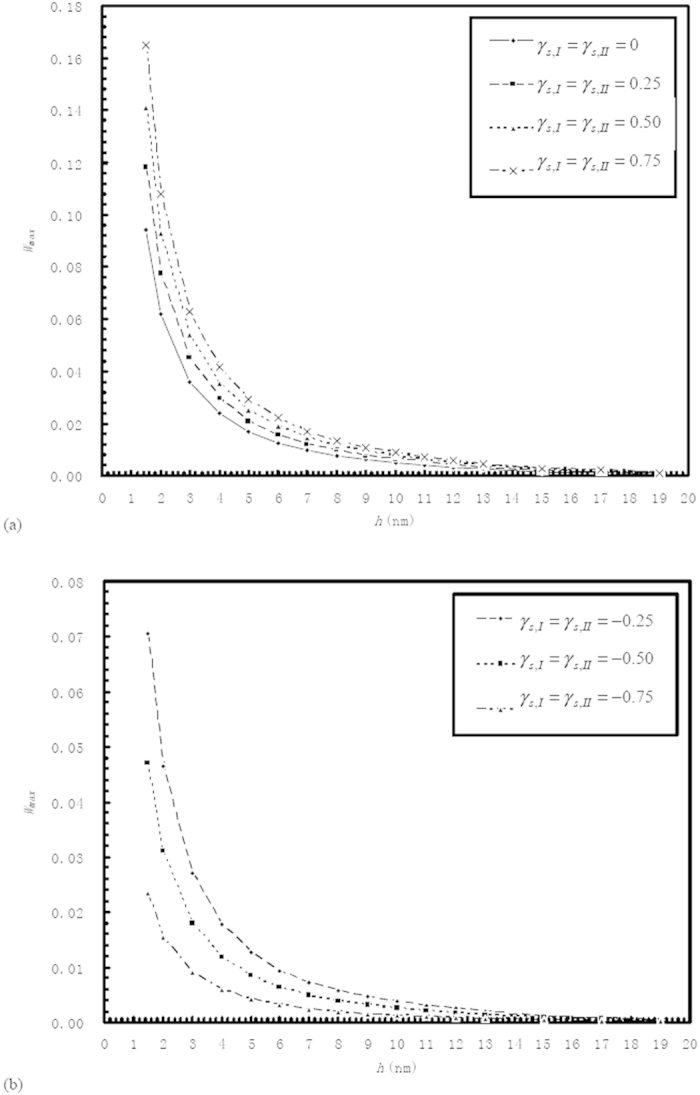
Plots of the value of the dimensionless *maximum* load 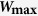 *carried by the bearing (in the optimum condition)* against the film thickness *h* for different values of 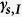 and 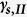 when the interaction between the confined fluid and the wall in the “a_1_” subzone is medium-level. 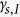
 *and* 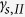 *are respectively the relative slip amounts of the confined fluid in the “I” and “II” subzones.* Description: For given values of 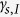 and 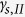, the film thickness reduction is shown to significantly increase the value of 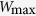 especially for very low film thicknesses.

Figure 4(a) plots the values of the dimensionless maximum load 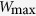 carried by the bearing (in the optimum condition) against the film thickness *h* for different positive values of 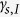 and 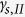 when the interaction between the confined fluid and the wall in the “a_1_” subzone is medium-level. Attend that in this figure 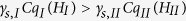. It means that the interfacial slippage in Fig. 4(a) is helpful for the generation of the load-carrying capacity of the bearing (according to Eq. (6)). This result is clearly shown by Fig. 4(a). The interfacial slippage in Fig. 4(a) can be attained when the fluid slippage is present at the whole stationary wall surface but absent at the whole moving wall surface (because of the strong interaction and consequently the high interfacial shear strength between the confined fluid and the moving wall). The mechanism of the increase of the load-carrying capacity of the bearing by the interfacial slippage in Fig. 4(a) is that for a given film thickness, the fluid mass flow rate in the Couette flow in the “I” subzone (into the bearing) is greater than that in the “II” subzone (out of the bearing) because 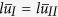 but the density of the confined fluid in the “I” subzone is higher than that in the “II” subzone, this mass flow rate difference is increased with the interfacial slippage, for maintaining the flow continuity in the bearing, higher pressures must be generated respectively in the “I” and “II” subzones to produce the corresponding Poiseuille flows to balance the total mass flow rate through the bearing when the interfacial slippage is increased, thus the load-carrying capacity of the bearing is increased with the interfacial slippage.

Figure 4(a) shows that for given values of 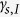 and 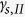, the film thickness reduction significantly increases the value of 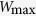 especially for very low film thicknesses. This is due to the increase of the non-continuum and physical adsorption effects of the confined fluid with the film thickness reduction. For very low film thicknesses, the strong non-continuum and physical adsorption effects of the confined fluid can greatly increase the load-carrying capacity of the bearing^21^,^34^.

Figure 4(b) plots the values of the dimensionless maximum load 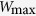 carried by the bearing against the film thickness *h* for different negative values of 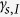 and 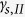 when the interaction between the confined fluid and the wall in the “a_1_” subzone is medium-level. The interfacial slippage in Fig. 4(b) is shown to be harmful for the generation of the load-carrying capacity of the bearing. It may be present when the fluid slippage occurs at the whole moving wall surface because of the weak interaction and then the low interfacial shear strength between the fluid and the moving wall. The mechanism of the interfacial slippage influence on the load-carrying capacity of the bearing in Fig. 4(b) is similar as in Fig. 4(a). In Fig. 4(b), for a given film thickness and negative values of 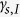 and 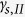, when the interfacial slippage is increased in both the bearing inlet and outlet zones, the (positive) difference between the fluid mass flow rate in the Couette flow in the “I” subzone and that in the “II” subzone is reduced because the entraining velocities 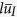 and 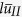 both are reduced 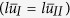, for maintaining the flow continuity in the bearing, lower pressures are required to be developed respectively in the “I” and “II” subzones to produce the corresponding Poiseuille flows to balance the total mass flow rate through the bearing, thus the load-carrying capacity of the bearing is reduced with the increase of the interfacial slippage. Again, for given values of 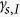 and 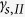, the film thickness reduction significantly increases the value of 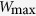 especially for very low film thicknesses.

**Figure 5:**
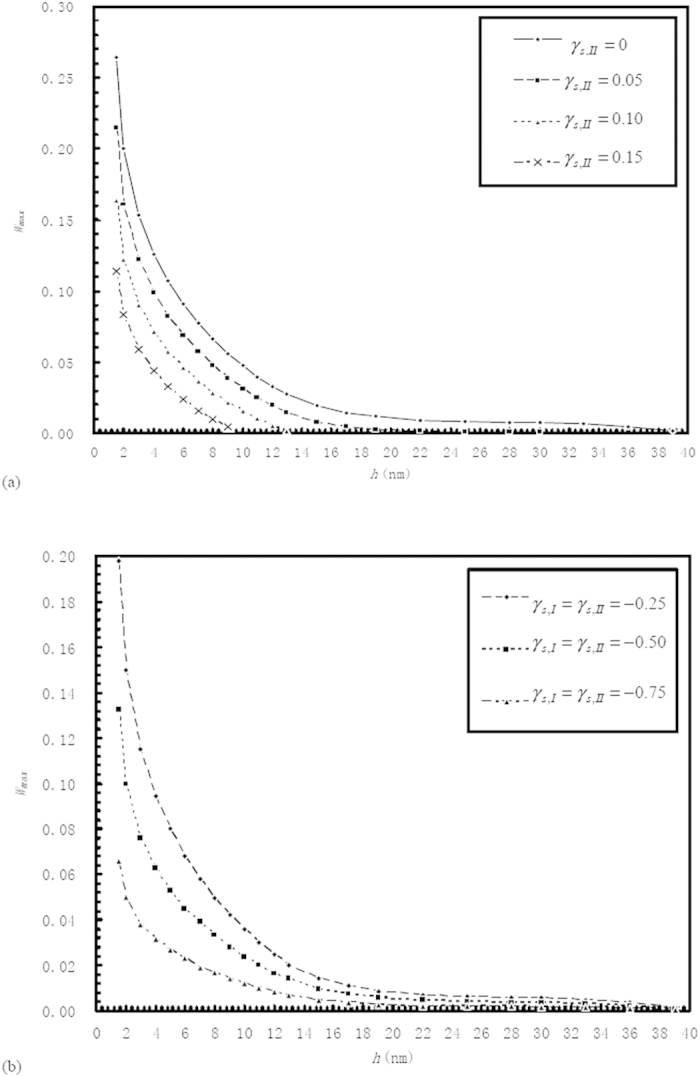
Plots of the value of the dimensionless *maximum* load 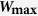 *carried by the bearing (in the optimum condition)*against the film thickness *h* for different values of 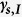 and 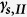 when the interaction between the confined fluid and the wall in the “a_1_” subzone is relatively strong. (**a**) 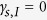. 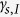 *and* 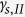 *are respectively the relative slip amounts of the confined fluid in the “I” and “II” subzones.* Description: For given values of 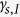 and 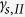, the film thickness reduction is shown to significantly increase the value of 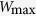 especially for very low film thicknesses. The comparison between Figs 4(a) and 5(a) as well as the comparison between Figs 4(b) and 5(b) show that for a given film thickness *h* and given values of 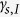 and 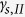, the increase of the interaction strength between the confined fluid and the wall in the “a_1_” subzone significantly increases the value of 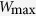 especially when the film thickness is low.

Figure 5(a) plots the values of the dimensionless maximum load 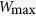 carried by the bearing against the film thickness *h* for 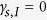 and different positive values of 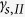 when the interaction between the confined fluid and the wall in the “a_1_” subzone is relatively strong. The interfacial slippage in Fig. 5(a) is harmful for the generation of the load-carrying capacity of the bearing. It may be present when the fluid slippage is only present at the stationary wall surface in the “II” subzone because of the weak fluid-wall interaction and then the low fluid-wall interfacial shear strength there. In Fig. 5(a), for a given film thickness, the interfacial slippage increase enhances the entraining velocity 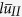 and then the fluid mass flow rate in the Couette flow in the “II” subzone, for maintaining the flow continuity in the bearing, lower pressures are required to be generated respectively in the “I” and “II” subzones to reduce the magnitudes of the Poiseuille flows in these two subzones to balance the total mass flow rate through the bearing when the interfacial slippage is increased, thus the load-carrying capacity of the bearing is reduced with the interfacial slippage increase.

Figure 5(b) plots the values of the dimensionless maximum load 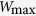 carried by the bearing against the film thickness *h* for different negative values of 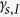 and 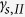 when the interaction between the confined fluid and the wall in the “a_1_” subzone is relatively strong. Similar as in Fig. 4(b), the interfacial slippage in this figure is harmful for the generation of the load-carrying capacity of the bearing.

The comparison between Figs 4(a) and 5(a) as well as the comparison between Figs 4(b) and 5(b) show that for a given film thickness *h* and given values of 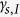 and 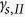, the increase of the interaction strength between the confined fluid and the wall in the “a_1_” subzone significantly increases the value of 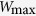 especially when the film thickness is low. This is due to the increasing non-continuum and physical adsorption effects of the confined fluid in the “I” subzone, which is very strong for low lubricating film thicknesses.

References 21 and 34 showed the carried loads of a nano step bearing in the optimum condition when the interfacial slippage was respectively artificially introduced and absent, by using the physical adsorption data of the confined fluid very close to those used in the present study. The comparison between the carried loads of that bearing and those of the present bearing shows that in the same operating condition, the maximum carried load of the present bearing (in the optimum condition) is comparable to that of a nano step bearing, although it is a bit lower than the latter. The advantages of the present bearing may be obvious: a bearing with a comparable load-carrying capacity but simple geometries and structures and the ensuing possible low costs of manufacturing.

### Results for an exemplary bearing

For evaluating the performance of the present bearing, the results for an exemplary bearing are here given. The total width 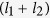 of this bearing is 20 *μm*. The used lubricant is a common oil with short-chain molecules and its viscosity at ambient condition is 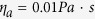. The stationary wall surface in the “II” subzone is covered with a hydrophobic coating such as the material of PTFE (Polytetrafluoroetylene) so that the interaction between this wall surface and the lubricant is relatively weak; The physical adsorption data of the confined lubricant in the “II” subzone are assumed as those shown in Tables 1, 2, 3, and 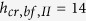 *nm*. The stationary wall surface in the “I” subzone is covered with a hydrophilic coating such as the material of TiO_2_ so that the interaction between this wall surface and the lubricant is medium-level; The physical adsorption data of the confined lubricant in the “I” subzone are assumed as those shown in Tables 1, 2, 3 for the medium interaction, and 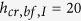 *nm*. In this case, for 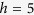 *nm*, the optimum value (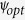) of *ψ* for the maximum load-carrying capacity of the bearing is 1.53. For realizing this condition, here 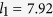 *μm* and 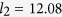 *μm*. If 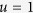 *mm*/*s* and 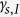 = 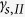 = 0.25, when 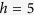 *nm*, the dimensional load (*w*) per unit contact length carried by the bearing in this optimum condition is 21.12 *N*/*m*.

## Conclusions

The paper proposes a novel nano bearing based on the different physical adsorptions of the lubricating film to the specific contact surfaces. The bearing is formed by two parallel smooth solid plane walls sliding against one another. Conventional hydrodynamic lubrication theory denied the formation of such a bearing.

Based on the flow factor approach model, an analysis was derived for the load-carrying capacity of this bearing. The condition for the formation of this bearing was derived to be: 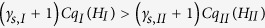. The optimum geometry for the maximum load-carrying capacity and the corresponding carried load of the bearing were also derived. It was found that the mechanism of the load-carrying capacity of this bearing may actually be the combined effects of the physical adsorption, non-continuum and interfacial slippage of the confined fluid; Any of these three effects is able to generate a significant load-carrying capacity of the bearing when the operating condition is appropriate. For low lubricating film thicknesses, the load-carrying capacity of the bearing is comparable to that of a nano step bearing for the same operating condition, because of the strong physical adsorption and non-continuum effects of the confined fluid. The study shows potential application values of this bearing in micro mechanical systems.

## Additional Information

**How to cite this article**: Zhang, Y. Novel nano bearings constructed by physical adsorption. *Sci. Rep.* **5**, 14539; doi: 10.1038/srep14539 (2015).

## Supplementary Material

Supplementary Information

## Figures and Tables

**Table 1:**
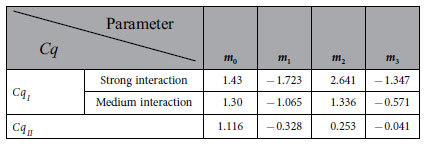
The values of the parameters in 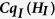 and 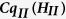
41.

**Table 2:**
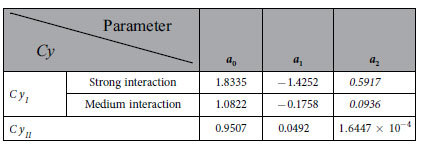
The values of the parameters in 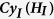 and 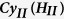
41.

**Table 3:**
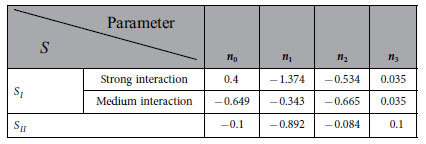
The values of the parameters in 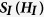 and 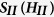 41.
