# Supplementary material for: Novel nano bearings constructed by physical adsorption
Source: Sci Rep. 2015 Sep 28;5:14539. doi: 10.1038/srep14539 (PMC4585955; doi:10.1038/srep14539)
Supplement: Supplementary Information [file srep14539-s1.pdf]

A quantitative comparison between the flow factor approach model and the molecular dynamics simulation results for the flow of a confined molecularly thin fluid film

Yongbin Zhang

Theoretical and Computational Fluid Dynamics

ISSN 0935-4964
Volume 29
Number 3

Theor. Comput. Fluid Dyn. (2015)
29:193-204
DOI 10.1007/s00162-015-0348-7

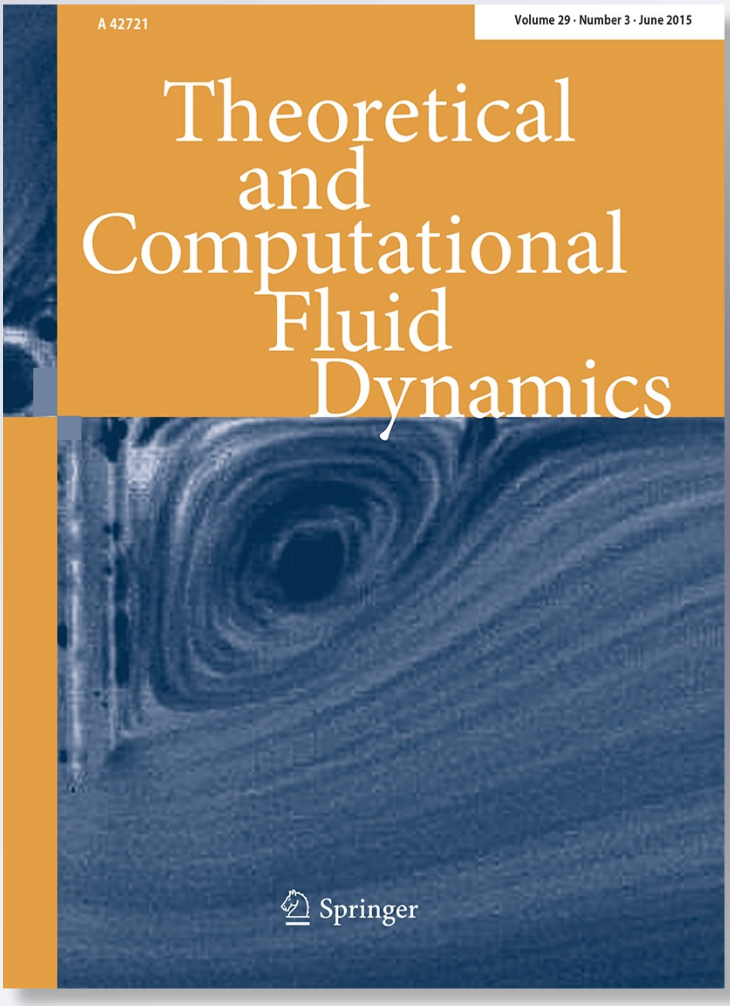

Springer

Your article is protected by copyright and all rights are held exclusively by Springer-Verlag Berlin Heidelberg. This e-offprint is for personal use only and shall not be self-archived in electronic repositories. If you wish to self-archive your article, please use the accepted manuscript version for posting on your own website. You may further deposit the accepted manuscript version in any repository, provided it is only made publicly available 12 months after official publication or later and provided acknowledgement is given to the original source of publication and a link is inserted to the published article on Springer's website. The link must be accompanied by the following text: "The final publication is available at link.springer.com”.

Springer

Author's personal copy

Theor. Comput. Fluid Dyn. (2015) 29:193–204
DOI 10.1007/s00162-015-0348-7

ORIGINAL ARTICLE

Yongbin Zhang

A quantitative comparison between the flow factor approach model and the molecular dynamics simulation results for the flow of a confined molecularly thin fluid film

Received: 8 November 2014 / Accepted: 1 April 2015 / Published online: 17 April 2015
© Springer-Verlag Berlin Heidelberg 2015

Abstract Quantitative comparisons were made between the flow factor approach model and the molecular dynamics simulation (MDS) results both of which describe the flow of a molecularly thin fluid film confined between two solid walls. Although these two approaches, respectively, calculate the flow of a confined molecularly thin fluid film by different ways, very good agreements were found between them when the Couette and Poiseuille flows, respectively, calculated from them were compared. It strongly indicates the validity of the flow factor approach model in modeling the flow of a confined molecularly thin fluid film.

Keywords Molecularly thin fluid film · Couette flow · Poiseuille flow · Model

1 Introduction

Modeling the flow of a confined molecularly thin fluid film is an important issue for both engineering application and academic researches. In the previous studies, such a modeling mainly relied on molecular dynamics simulation (MDS). Bitsanis et al. [1,2], respectively, modeled the Couette and Poiseuille flows of molecularly thin fluid films confined between two solid walls by MDS. They showed the obvious distortions of the velocity profiles of the confined film across the film thickness compared to the conventional theory description when the film thickness was only several times of the fluid molecule diameter, because of the local film density variations across the film thickness. Somers and Ted Davis [3] as well as Jabbarzadeh et al. [4] obtained the similar results for the Couette flow of the confined molecularly thin fluid film. Takaba et al. [5] modeled the Poiseuille flow of a molecularly thin fluid film, respectively, confined between two solid walls and in a microcylindrical pore by MDS. They showed that when the film thickness was on the same scale of the fluid molecule diameter, the velocity profile across the film thickness of the confined film in the Poiseuille flow was actually distorted compared to the conventional theory description, and its velocity magnitude deviated from the result calculated from the conventional theory and strongly depended on the interaction strength between the confined film and the solid wall. They showed that a stronger film–wall interaction resulted in a smaller magnitude of the velocity of the confined film and related the flow of the confined film to the local density variations across the film thickness.

The wall roughness effect on the flow in nanochannels was also studied by MDS. Sofos et al. [6] and Kasiteropoulou et al. [7] showed that the increase in the height of the atomistic-scale protrusion on the solid wall reduced the magnitude of the flow velocity of the fluid in the Poiseuille flow in nanochannels and also reduced the slipping velocity of the fluid at the rough wall. Sofos et al. [8] showed that the wall surface roughness had a significant effect on the shear viscosity of the fluid film confined in a nanochannel.

Communicated by O. Zikanov.

Y. Zhang (✉)
College of Mechanical Engineering, Changzhou University, Changzhou, Jiangsu Province, China
E-mail: engmech1@sina.com

Author's personal copy

By the dissipative particle dynamics approach, Kasiteropoulou et al. [9] studied the effects of the wall–fluid interaction, the wall density, and the cutoff circle radius on the flow velocity of the fluid in the Poiseuille flow in a nanochannel. They showed that the increase in the repulsion between the wall and the fluid, the increase in the wall density, or the reduction in the cutoff circle radius all significantly increase the magnitude of the fluid flow velocity. This may correspond to the weakening of the wall–fluid interaction strength which resulted in the increase in the magnitude of the flow velocity of the confined film.

Although there have been various studies on the fluid flow in microscale or nanoscale channels by molecular dynamics simulation or other similar simulations, those studies failed to provide a model depicting the fluid flow in a nanochannel which can be efficiently applied in an engineering modeling.

According to the rheological properties of confined molecularly thin fluid films, on the equilibrium level, Zhang [10] and Zhang and Lu [11] proposed a model called as "the flow factor approach" describing the flow of confined molecularly thin fluid films. The velocity profiles across the film thickness of the confined film, respectively, in the Couette and Poiseuille flows can also be calculated from that model.

In this paper, quantitative comparisons were extensively made between the flow factor approach model and the MDS results when they both calculated the velocity distributions across the film thickness of the confined molecularly thin fluid film, respectively, in the Couette and Poiseuille flows. In the comparisons, the operating conditions were the same in both the calculations, and they were isothermal with perfectly smooth wall surfaces. It was found that the agreements between the flow factor approach model and the MDS results are very good. The results in this paper strongly indicate the validity of the flow factor approach model in modeling the flow of a confined molecularly thin fluid film.

2 The flow factor approach model

On the equilibrium level, Zhang [10] and Zhang and Lu [11] analyzed the flow of a molecularly thin fluid film confined between two solid walls from micromechanics. They treated the fluid molecule as a rigid ball and considered the fluid inhomogeneity and discontinuity across the film thickness. In Zhang's analysis [10], the fluid inhomogeneity across the film thickness was characterized by the variations across the film thickness of both the fluid viscosity and the separation between the neighboring fluid molecules, and the fluid discontinuity across the film thickness was characterized by the varying separations between the neighboring fluid molecules across the film thickness. Both the fluid inhomogeneity and discontinuity across the film thickness were intimately related to the confined film–solid wall interactions. This section briefly describes Zhang's analytical model and his obtained results [10].

Figure 1a shows the modeled system in Zhang's analysis [10]. By an equivalent transformation method, a molecularly thin fluid film confined between two parallel solid plane walls can be treated as shown in Fig. 1a [12]. In Fig.1a, the confined film is ordered to the solid wall in the normal direction because of the film–wall interaction, and the momentum transfer mainly occurs in the direction normal to the solid wall within the confined film when the solid walls slide against one another. Both the local viscosity and the separation between the neighboring fluid molecules across the film thickness are varied dependent on the distance from the solid wall. They play important roles in the momentum transfer across the film thickness.

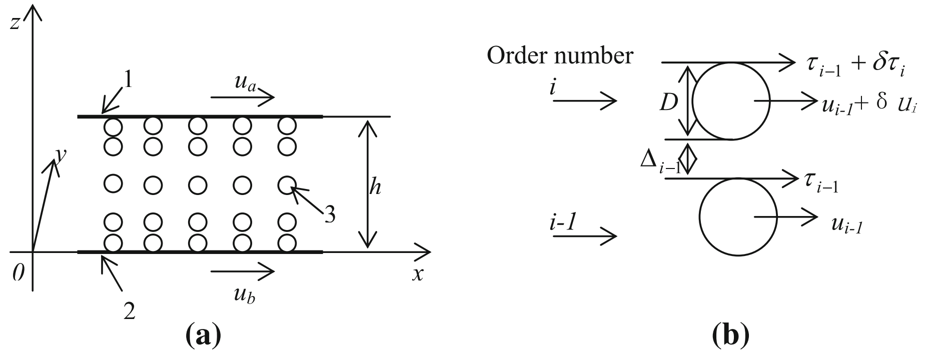

Fig. 1 A molecularly thin fluid film confined between two solid walls [10]. **a** Magnified low fluid film thickness, ordered (non-continuum) fluids across the film thickness. **b** Two exemplary magnified fluid molecules across the film thickness. 1—*upper* contact surface, 2—*lower* contact surface, 3—ordered fluids. u_a and u_b are, respectively, the speeds of the *upper* and *lower* contact surfaces

Author's personal copy

Figure 1b selects out two neighboring fluid molecules across the film thickness for analysis [10]. In Fig. 1b, only the viscous shear stress occurs on the fluid molecule, and it is [10]:

$$\tau_{i-1} = \frac{\eta_{\text{line},i-1}\delta u_i}{\Delta_{i-1}} \tag{1}$$

where i and $(i-1)$ are, respectively, the order numbers of these two molecules across the film thickness; δu_i, $\eta_{\text{line},i-1}$, and Δ_{i-1} are, respectively, the velocity difference, the local viscosity, and the separation between these two molecules; and τ_{i-1} is the shear stress acting on the $(i-1)$th molecule across the film thickness. The momentum equilibrium equation of the upper molecule is [10]:

$$\delta\tau_i = \frac{\partial p}{\partial x} D \tag{2}$$

where $\delta\tau_i$ is the shear stress difference between these two molecules, D is the fluid molecule diameter, p is the pressure of the confined film, and x is the coordinate in the direction of the confined film flow (shown in Fig. 1a).

Here, all the mentioned phenomenological parameters such as the pressure, shear stress, and local viscosity of the confined film as well as the velocity difference and the separation between the neighboring film molecules are ensemble averaged over time and not varied with time for a steady-state operating condition. The definitions of these phenomenological parameters can be found in a lot of MDS such as in Refs. [1–9].

The velocity of the ith fluid molecule across the film thickness (for $i = 1, 2, \ldots, (n-1)$, n is the number of the fluid molecules across the film thickness) was derived to be [10]:

$$\begin{aligned} u_i = \bar{u}_b + \frac{i(\bar{u}_a - \bar{u}_b)(\Delta_l/\eta_{\text{line},l})_{\text{avr},i}}{(n-1)(\Delta_l/\eta_{\text{line},l})_{\text{avr},n-1}} + Di\frac{\partial p}{\partial x}(l\Delta_{l-1}/\eta_{\text{line},l-1})_{\text{avr},i} \\ \times \left[1 - \frac{(\Delta_l/\eta_{\text{line},l})_{\text{avr},i}(l\Delta_{l-1}/\eta_{\text{line},l-1})_{\text{avr},n-1}}{(\Delta_l/\eta_{\text{line},l})_{\text{avr},n-1}(l\Delta_{l-1}/\eta_{\text{line},l-1})_{\text{avr},i}}\right] \end{aligned} \tag{3}$$

where $\bar{u}_a$ is the velocity of the $(n-1)$th fluid molecule across the film thickness, which is on the upper solid wall; $\bar{u}_b$ is the velocity of the 0th fluid molecule across the film thickness, which is on the lower solid wall,

$$(\Delta_l/\eta_{\text{line},l})_{\text{avr},i} = \frac{\sum_{l=0}^{i-1}\Delta_l/\eta_{\text{line},l}}{i} \tag{4}$$

and

$$(l\Delta_{l-1}/\eta_{\text{line},l-1})_{\text{avr},i} = \frac{\sum_{l=1}^{i} l\Delta_{l-1}/\eta_{\text{line},l-1}}{i} \tag{5}$$

According to Eq. (3), the present study considers the possible slippage of the confined film over the entrapping solid surfaces. This treatment fits the findings of the slippage of the confined molecularly thin film over the entrapping solid walls as have been found in various MDS and experiments [1–5]. Indeed, this interfacial slippage of the confined film may depend on the confined film–solid surface interfacial shear strength [13]. It occurs when the shear stress of the confined film exceeds the interfacial shear strength, so that the velocity ($\bar{u}_a$ or $\bar{u}_b$) of the film molecule at the solid surface is different from the corresponding solid surface speed (u_a or u_b). It does not occur when the shear stress of the confined film is below the interfacial shear strength, so that the velocity of the film molecule at the solid surface is equal to the corresponding solid surface speed. The interfacial slippage of the confined film especially occurs when the confined film has a weak interaction with the entrapping solid surface, because of the resulting low interfacial shear strength.

In the present study, it is assumed that the separation between the neighboring fluid molecules across the film thickness is symmetrical with respect to the median plane of the confined film [10]. As Zhang did [10], it is here taken that $\Delta_{i+1}/\Delta_i = q_0 > 1$ and $\eta_{\text{line},i}/\eta_{\text{line},i+1} = q_0^m > 1$ (for $i = 0, 1, \ldots, (n-1)/2 - 2$). Here, q_0 and m are, respectively, constant, and n is the number of the fluid molecules across the film thickness and is an odd number ($n \geq 5$). Nevertheless, the values of Δ_{i+1}/Δ_i and $\eta_{\text{line},i}/\eta_{\text{line},i+1}$ both may be varied across the film thickness, the model takes q_0 as the average value of Δ_{i+1}/Δ_i (for $i = 0, 1, \ldots, (n-1)/2 - 2$). These treatments are well supported by the MDS results which showed varying local densities of the confined film across the film thickness and the corresponding varying local separations between the neighboring fluid molecules across the film thickness [1–5].

Author's personal copy

In the present model, the fluid film–wall interactions are considered, reflected by both the values of q_0 and Δ_{im}/D. A higher value of q_0 but a lower value of Δ_{im}/D indicates a stronger fluid film–wall interaction. The values of q_0 and Δ_{im}/D both can be determined by fitting the ab initio calculation results with the present model.

In the present model, detailed microscopic acting factors ever taken in the molecular dynamics simulation such as the intermolecular forces, the wall density, and the cutoff circle radius are not used. The effects of those factors on the fluid flow are instead studied here by using the phenomenological parameters of the fluid film pressure, the fluid film shear stress, the averaged separations between the neighboring fluid molecules across the film thickness, the ratios between these separations, and the inhomogeneous viscosities across the film thickness. In the present model, modulating the values of the parameters of the number (n) of the fluid molecules across the film thickness, the dimensionless separation (Δ_{im}/D) between the neighboring fluid molecules across the film thickness in the middle plane of the confined film, the averaged molecule separation ratio (q_0) across the film thickness, and the viscosity inhomogeneity index (m) across the film thickness corresponds to different fluid film thicknesses and different wall–fluid interaction strengths.

In the present study, the dimensionless parameters are defined as follows:

$$U_i = \frac{u_i}{\bar{u}_a - \bar{u}_b}, \quad \bar{U}_b = \frac{\bar{u}_b}{\bar{u}_a - \bar{u}_b}, \quad Z = \frac{z}{D}$$

2.1 Couette flow

When $\partial p/\partial x = 0$, the confined film is in the Couette flow. For this case, according to Eq. (3), the dimensionless velocity of the fluid molecule is finally written as [10]:

$$U_i = \begin{cases} \bar{U}_b + \dfrac{q_0^{(m+1)i} - 1}{2\left[q_0^{\frac{(n-1)(m+1)}{2}} - 1\right]}, & \text{for} \quad 0 \le i \le (n-1)/2 \\[2ex] \bar{U}_b + \dfrac{2q_0^{\frac{(n-1)(m+1)}{2}} - q_0^{(m+1)(n-1-i)} - 1}{2\left[q_0^{\frac{(n-1)(m+1)}{2}} - 1\right]}, & \text{for} \quad (n-1)/2 < i \le (n-1) \end{cases} \tag{6}$$

2.2 Poiseuille flow

The last term on the right-hand side of Eq. (3) is the poiseuille flow. The dimensionless velocity of the fluid molecule owing to the Poiseuille flow is [10]:

$$U_i = \Gamma_p \left[G_1(i) - \frac{G_0(i)G_1(n-1)}{G_0(n-1)}\right] \tag{7}$$

where $\Gamma_p = D\Delta_{im}(\partial p/\partial x)/[\eta_{\text{line},im}(\bar{u}_a - \bar{u}_b)]$, $G_0(i)$ is:

$$G_0(i) = \begin{cases} \dfrac{q_0^{(m+1)i} - 1}{q_0^{\frac{(n-1)(m+1)}{2}} - q_0^{\frac{(n-3)(m+1)}{2}}}, & \text{for} \quad 0 \le i \le \frac{n-1}{2} \\[2ex] \dfrac{q_0^{\frac{(n-1)(m+1)}{2}} - 1}{q_0^{\frac{(n-1)(m+1)}{2}} - q_0^{\frac{(n-3)(m+1)}{2}}} + \dfrac{q_0^{\left(\frac{n+1}{2} - i\right)(m+1)} - q_0^{m+1}}{1 - q_0^{m+1}}, & \text{for} \quad \frac{n-1}{2} < i \le n-1 \end{cases} \tag{8}$$

and $G_1(i)$ is:

$$G_1(i) = \begin{cases} \dfrac{1}{q_0^{\frac{(n-3)(m+1)}{2}}}\left[\dfrac{1 - q_0^{(m+1)i}}{(q_0^{m+1} - 1)^2} + \dfrac{i q_0^{(m+1)i}}{q_0^{m+1} - 1}\right], & \text{for} \quad 0 \le i \le \frac{n-1}{2} \\[2ex] \dfrac{1}{q_0^{\frac{(n-3)(m+1)}{2}}}\left[\dfrac{1 - q_0^{\frac{(n-1)(m+1)}{2}}}{(q_0^{m+1} - 1)^2} + \dfrac{(n-1)q_0^{\frac{(n-1)(m+1)}{2}}}{2(q_0^{m+1} - 1)}\right] - \dfrac{(n-1)\left[q_0^{(\frac{n+1}{2} - i)(m+1)} - q_0^{m+1}\right]}{2(q_0^{m+1} - 1)} \\ \quad + \dfrac{1 - q_0^{(\frac{n-1}{2} - i)(m+1)}}{[q_0^{-(m+1)} - 1]^2} + \dfrac{(i - \frac{n-1}{2})q_0^{(\frac{n-1}{2} - i)(m+1)}}{q_0^{-(m+1)} - 1}, & \text{for} \quad \frac{n-1}{2} < i \le n-1 \end{cases} \tag{9}$$

Here, $im = (n-1)/2$.

Author's personal copy

3 Comparison of the flow factor approach model with the MDS results

3.1 For the Couette flow

Figure 2a–c shows the velocity distributions across the film thickness of the confined molecularly thin fluid film in the Couette flow calculated from the MDS by Jabbarzadeh et al. [4] for different film–wall interaction

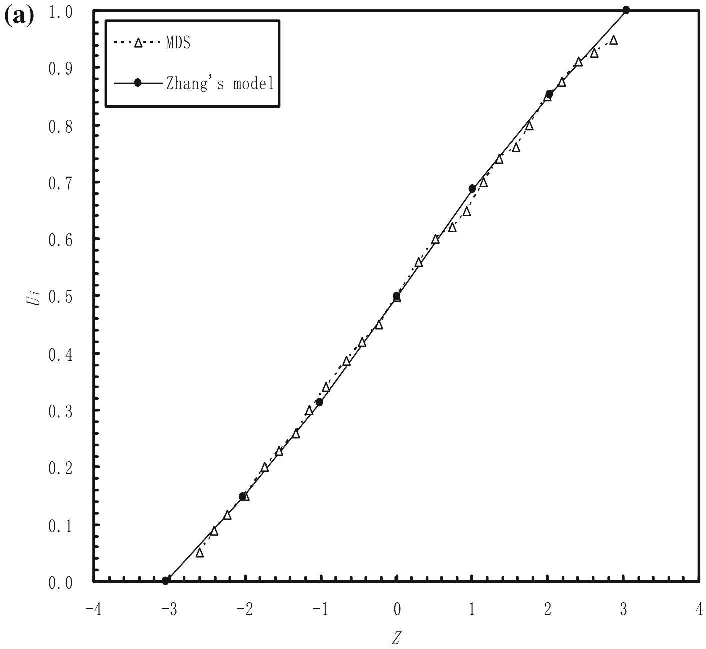

In Zhang's model, n=7, $\Delta_{im}/D = 0.017$, m=3.0, and $q_0 = 1.03$.

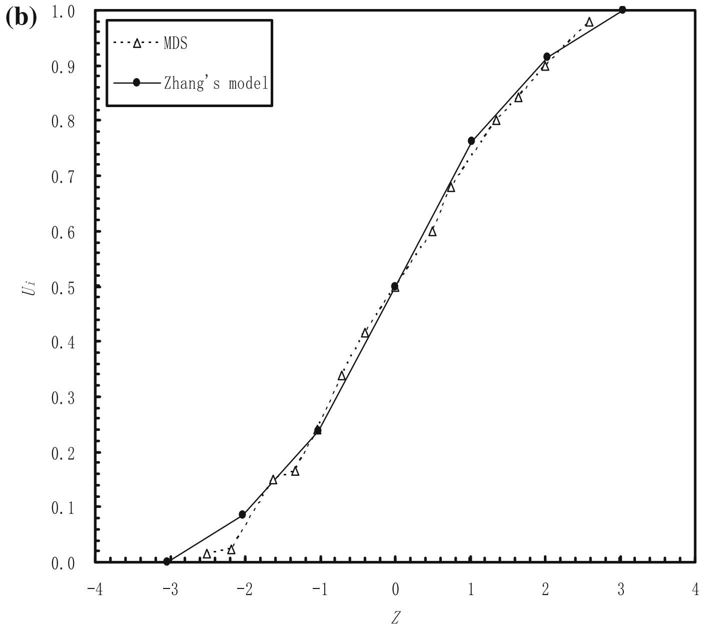

In Zhang's model, n=7, $\Delta_{im}/D = 0.016$, m=3.0, and $q_0 = 1.1$.

Fig. 2 Comparison of the velocity profile across the film thickness of a molecularly thin fluid film in the Couette flow confined between two solid walls obtained from the molecular dynamics simulation (MDS) by Jabbarzadeh et al. [4] with that calculated from Zhang's model [10]. In MDS, $h = 7D$, the shear rate was $0.2(\varepsilon/MD^2)^{1/2}$. **a** $\varepsilon_w = 1.0\varepsilon$, **b** $\varepsilon_w = 2.0\varepsilon$, **c** $\varepsilon_w = 3.0\varepsilon$

Author's personal copy

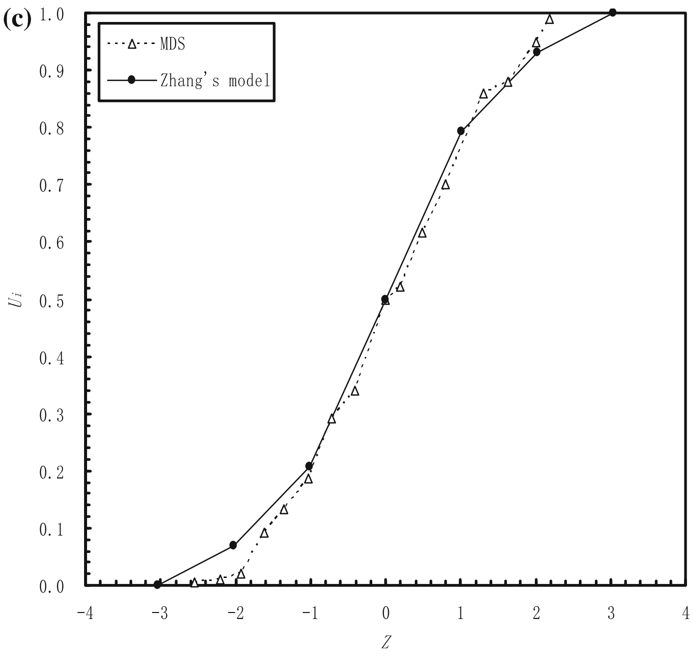

In Zhang's model, n=7, $\Delta_{im}/D = 0.014$, m=3.0, and $q_0 = 1.2$.

Fig. 2 continued

strengths when the wall separation h was fixed as $7D$, and the shear rate was $0.2(\varepsilon/MD^2)^{1/2}$. Here, ε is the interaction potential energy between the fluid atoms, ε_w is the interaction potential energy between the solid wall atom and the fluid atom, and M is the mass of the fluid molecule [4]. Jabbarzadeh et al. performed an NVT-ensemble simulation of the Couette flow of the fluid confined between two parallel solid plane walls when the walls slid against one another, by using the Lennard-Jones potential to model both the interaction between the fluid atoms and the interaction between the fluid atom and the wall atom [4]. They used different values of ε_w to simulate the different interaction strengths between the fluid atom and the wall atom, and calculated the time-averaged velocities of the fluid molecules across the wall gap for different wall separations and different ε_w values.

The MDS results in Fig. 2a–c are compared with the calculation results from the flow factor approach model for the same operating conditions. In the comparisons, not only the variation trends of the U_i versus Z curve but also the magnitudes of the velocities of the confined film molecules fairly agree with one another. These figures show that when the value of ε_w, i.e., the interaction strength between the confined film and the solid wall, is increased, the value of Δ_{im}/D is significantly reduced, but the value of q_0 is significantly increased according to the flow factor approach model. This shows that both the solidification and the inhomogeneity across the film thickness of the confined film were significantly increased with the increase in the interaction strength between the confined film and the solid wall. Such results are completely fitting the results obtained by Jabbarzadeh et al. [4] in the MDS of the local density variations across the film thickness of the confined film for different ε_w values.

Figure 3a–c shows the velocity distributions across the film thickness of the confined molecularly thin fluid film in the Couette flow calculated from the MDS by Jabbarzadeh et al. [4] for different film–wall interaction strengths when the wall separation h was fixed as $10D$ and the shear rate was $0.2(\varepsilon/MD^2)^{1/2}$. The results in these figures are similar as in Fig. 2a–c. Again, the flow factor approach model is shown to fairly agree with the MDS results. The comparisons between the results calculated from the flow factor approach model, respectively, in Figs. 2a–c and 3a–c show that for a given ε_w value, the increase in the wall separation h significantly increases Δ_{im}/D but reduces q_0. This shows that both the solidification and the inhomogeneity across the film thickness of the confined film are significantly reduced with the increase in the wall separation, and the rheological properties of the confined film are heavily dependent on the film–wall interaction, the effect of which is controlled by the film thickness.

Author's personal copy

3.2 For the Poiseuille flow

Figure 4a–d shows the distributions of $u_i/u_{\max}$ across the film thickness of the confined molecularly thin fluid film in the Poiseuille flow calculated from the MDS by Takaba et al. [5] and their comparisons with the results calculated from the flow factor approach model. Here, $u_{\max}$ is the maximum velocity of the confined

(a)

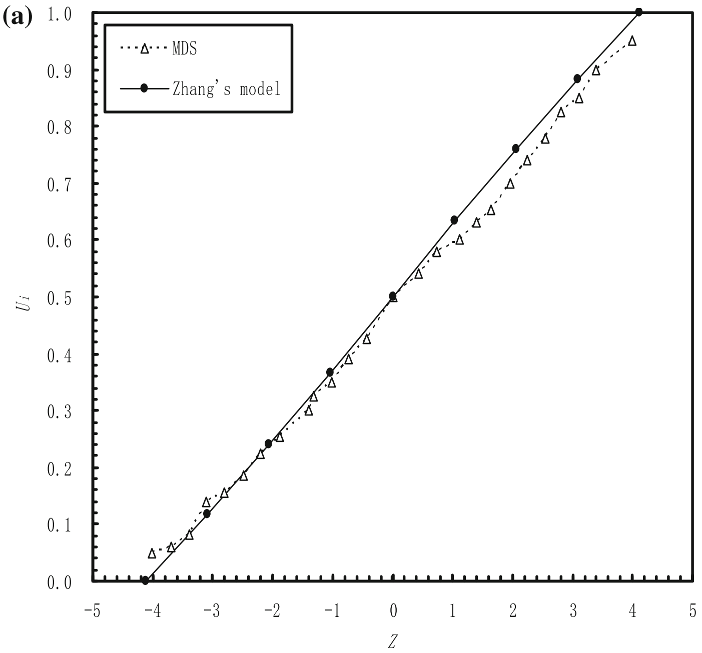

In Zhang's model, n=9, $\Delta_{im}/D = 0.03$, m=3.0, and $q_0 = 1.01$.

(b)

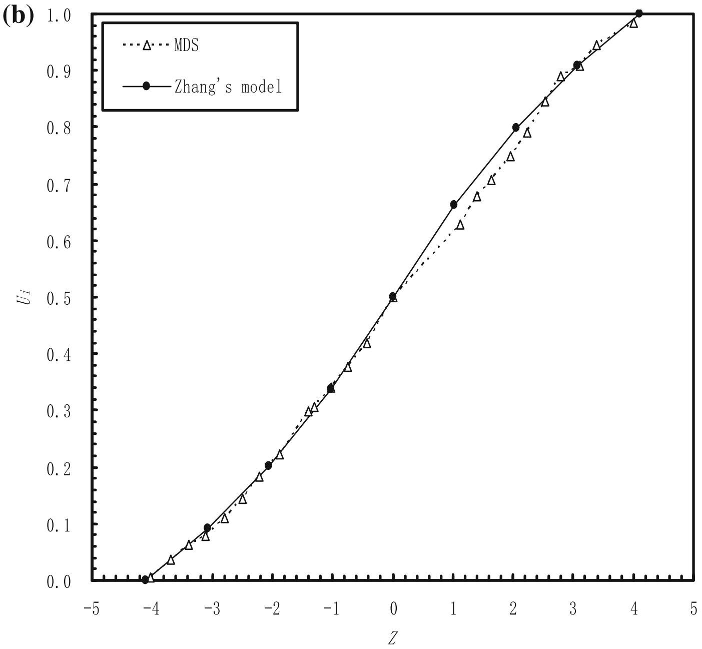

In Zhang's model, n=9, $\Delta_{im}/D = 0.026$, m=3.0, and $q_0 = 1.05$.

Fig. 3 Comparison of the velocity profile across the film thickness of a molecularly thin fluid film in the Couette flow confined between two solid walls obtained from the molecular dynamics simulation (MDS) by Jabbarzadeh et al. [4] with that calculated from Zhang's model [10]. In MDS, $h = 10D$, the shear rate was $0.2(\varepsilon/MD^2)^{1/2}$. **a** $\varepsilon_w = 1.0\varepsilon$. **b** $\varepsilon_w = 2.0\varepsilon$. **c** $\varepsilon_w = 3.0\varepsilon$

Author's personal copy

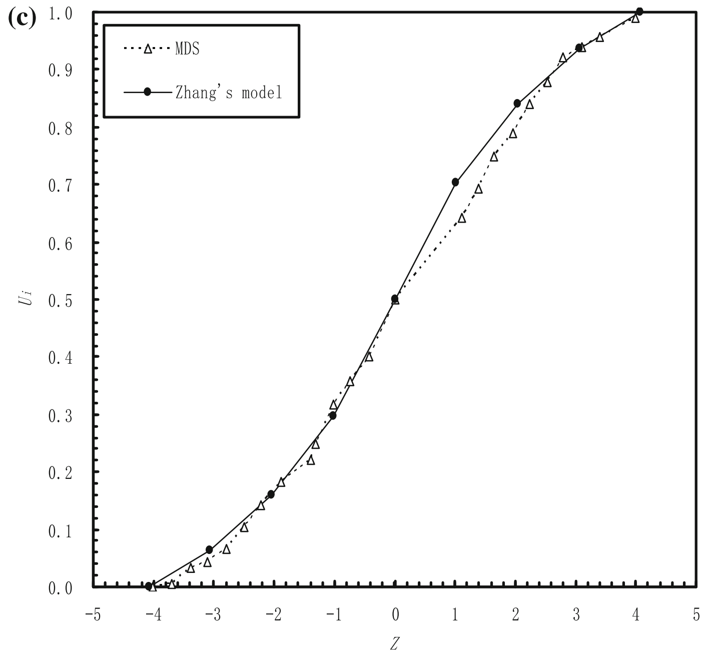

In Zhang's model, n=9, $\Delta_{im}/D = 0.023$, m=3.0, and $q_0 = 1.1$.

Fig. 3 continued

film across the film thickness. Takaba et al. simulated the Poiseuille flow of the fluid confined in microslit or cylindrical pores when the pressure gradient in the pore was generated and the system temperature was fixed, by using the Lennard-Jones potential to model both the interaction between the fluid atoms and the interaction between the fluid atom and the wall atom [5]. They obtained the time-averaged velocities of the fluid molecules across the wall gap for different wall separations (h or d) and different interaction potentials (ε_w) between the fluid atom and the wall atom.

Figure 4a–d shows that for the Poiseuille flow, the flow factor approach model also fairly agrees with the MDS results. The comparison between the results calculated from the flow factor approach model in Fig. 4a, b also shows that for a given ε_w value, the increase in the wall separation h significantly increases Δ_{im}/D but reduces q_0. The comparison between the results calculated from the flow factor approach model in Fig. 4c, d shows that for a given cylindrical pore diameter d, the increase in the interaction strength between the confined film and the wall significantly reduces Δ_{im}/D but increases q_0. This shows that both the solidification and the inhomogeneity across the film thickness of the confined film are heavily influenced by the film–wall interaction strength. Figure 4a–d shows that the Poiseuille flow of a confined molecularly thin fluid film is not only determined by the film thickness but also determined by Δ_{im}/D and q_0, both of which reflect the non-continuum effect of the confined film governed by the film–wall interaction. These results agree well with the viewpoint obtained in the MDS by Takaba et al. [5] that the Poiseuille flow of a confined molecularly thin fluid film was strongly influenced by the local density variation across the film thickness of the confined film.

For further examining the validity of the flow factor approach model for depicting the Poiseuille flow, the maximum velocities $u_{\max}$ of the confined films across the film thickness, respectively, calculated from the MDS and the flow factor approach model were compared. Here, the parameter R_u is defined as:

$$R_u = \frac{u_{\max}(h)}{u_{\max}(15.2D)} \tag{10}$$

Figure 5 shows the comparison of the values of R_u, respectively, calculated from the MDS by Takaba et al. [5], and the flow factor approach model for different h/D values. Considering the calculation errors, the values of R_u, respectively, calculated from these two approaches should be close.

In the present study, a direct comparison of the absolute values of $u_{\max}$ in the Poiseuille flow, respectively, calculated from the MDS by others and the flow factor approach model is difficult to make because of the unknown values of $\partial p/\partial x$ and $\eta_{\text{line},im}$ in the MDS, which are required for calculating $u_{\max}$ according to the flow factor approach model. This direct comparison is put as a task in the future.

Author's personal copy

(a)
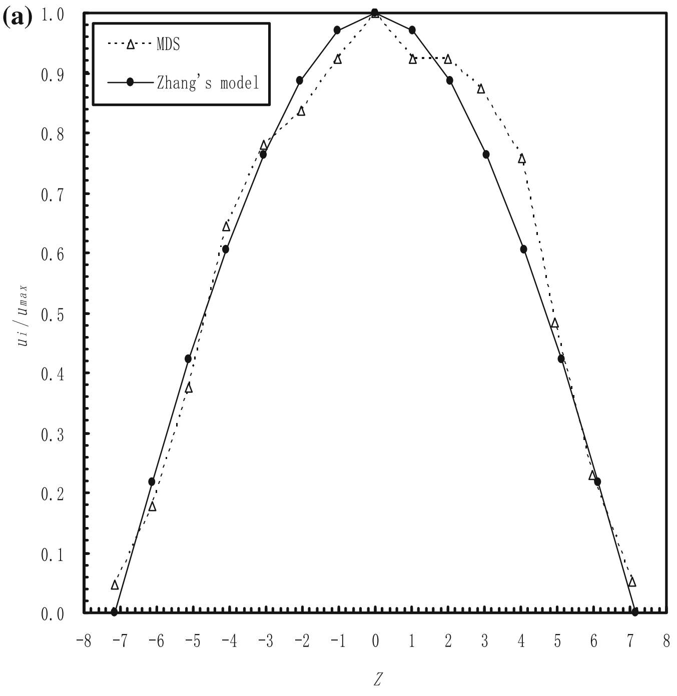

In MDS, $h = 15.2D$ and $\varepsilon_w = 0.202\varepsilon$ for the slit pore model;

In Zhang's model, n=15, $\Delta_{im} / D = 0.024$, m=3.0, and $q_0 = 1.025$.

(b)
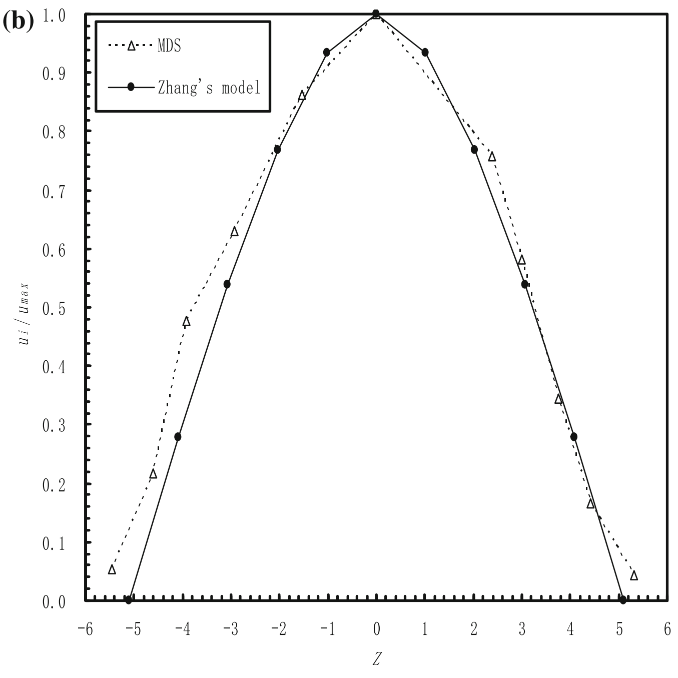

In MDS, $h = 11.2D$ and $\varepsilon_w = 0.202\varepsilon$ for the slit pore model;

In Zhang's model, n=11, $\Delta_{im} / D = 0.022$, m=3.0, and $q_0 = 1.05$.

Fig. 4 Comparison of the velocity profile across the film thickness of a molecularly thin fluid film in the Poiseuille flow confined between solid walls obtained from the molecular dynamics simulation (MDS) by Takaba et al. [5] with that calculated from Zhang's model [10]

Author's personal copy

(c)

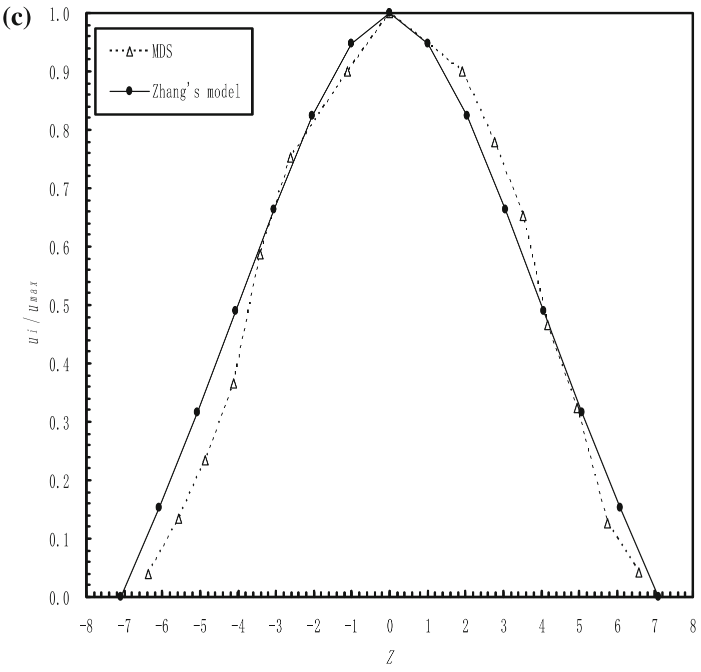

In MDS, $d = 15.2D$ and $\varepsilon_w = 0.605\varepsilon$ for the cylindrical pore model;
In Zhang's model, n=15, $\Delta_{im} / D = 0.017$, m=3.0, and $q_0 = 1.065$.

(d)

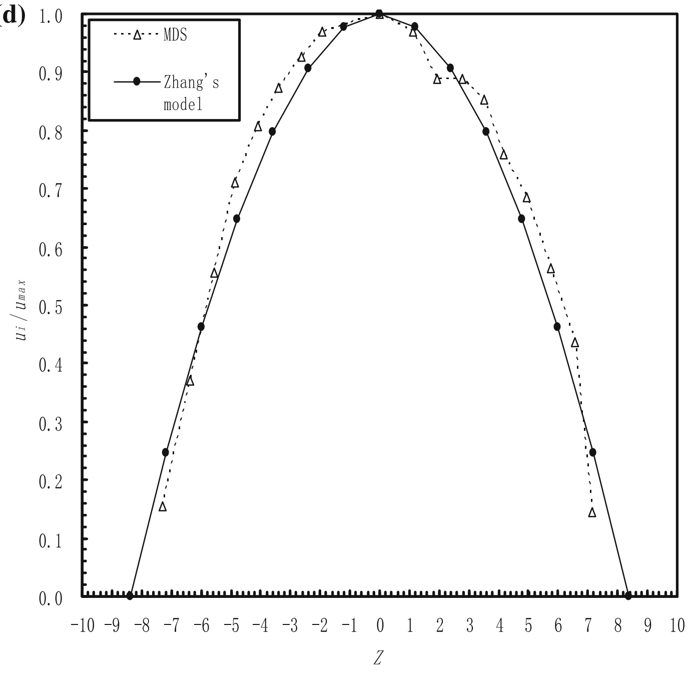

In MDS, $d = 15.2D$ and $\varepsilon_w = 0.202\varepsilon$ for the cylindrical pore model;
In Zhang's model, n=15, $\Delta_{im} / D = 0.2$, m=3.0, and $q_0 = 1.01$.

Fig. 4 continued

Author's personal copy

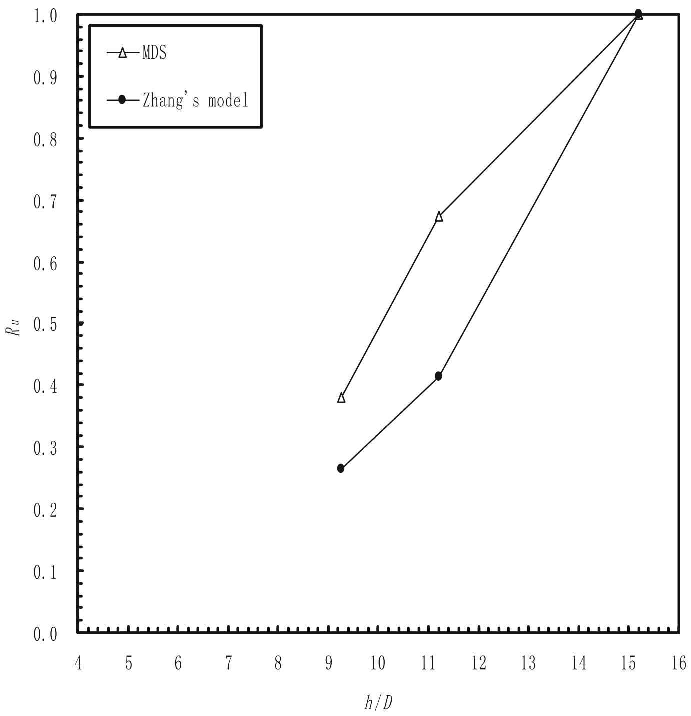

Fig. 5 Comparison of the values of R_u calculated from the molecular dynamics simulation (MDS) by Takaba et al. [5] for different h/D values with those calculated from Zhang's model [10]. The MDS results are for the slit pore model

4 Conclusions

The paper presents quantitative comparisons between the flow factor approach model and the molecular dynamics simulation results for the flow of a molecularly thin fluid film confined between two solid walls. Very good agreements were found between these two approaches for both the Couette and Poiseuille flows. The validity of the flow factor approach model was directly evidenced. This model is of significant interest for the modeling of the flow of a confined molecularly thin fluid film.

Conflict of interest I declare that this paper is an original work done by myself and has no conflict of interest with others.

References

1. Bitsanis, I, Magda, J.J., Tirrell, M., Davis, H.T.: Molecular dynamics of flow in micropores. J. Chem. Phys. **87**, 1733–1750 (1987)
2. Bitsanis, I., Vanderlick, T.K., Tirrell, M., Davis, H.T.: A tractable molecular theory of flow in strongly inhomogeneous fluids. J. Chem. Phys. **89**, 3152–3162 (1988)
3. Somers, S.A., Davis, H.T.: Microscopic dynamics of fluids confined between smooth and atomically structured solid surfaces. J. Chem. Phys. **96**, 5389–5407 (1992)
4. Jabbarzadeh, A., Atkinson, J.D., Tanner, R.I.: Rheological properties of thin liquid films by molecular dynamics simulations. J. Non-Newtonian Fluid Mech. **69**, 169–193 (1997)
5. Takaba, H., Onumata, Y., Nakao, S.: Molecular simulation of pressure-driven fluid flow in nanoporous membranes. J. Chem. Phys. **127**, 054703 (2007)
6. Sofos, D.F., Karakasidis, T.E., Liakopoulos, A.: Effects of wall roughness on flow in nanochannels. Phys. Rev. E. **79**, 026305 (2009)
7. Kasiteropoulou, D., Karakasidis, T.E., Liakopoulos, A: Mesoscopic simulation of fluid flow in periodically grooved microchannels. Comput. Fluids **74**, 91–101 (2013)
8. Sofos, D.F., Karakasidis, T.E., Liakopoulos, A.: Effect of wall roughness on shear viscosity and diffusion in nanochannels. Int. J. Heat Mass Transf. **53**, 3839–3846 (2010)
9. Kasiteropoulou, D., Karakasidis, T.E., Liakopoulos, A.: Dissipative particle dynamics investigation of parameters affecting planar nanochannel flows. Mater. Sci. Eng. B **176**, 1574–1579 (2011)
10. Zhang, Y.B.: Flow factor of non-continuum fluids in one-dimensional contact. Ind. Lubri. Trib. **58**, 151–169 (2006)

Author's personal copy

11. Zhang, Y.B., Lu, G.S.: Flow factor for molecularly thin fluid films in one-dimensional flow due to fluid discontinuity. J. Mol. Liq. **116**, 43–50 (2005)
12. Zhang, Y.B.: Flow factor approach to molecularly thin hydrodynamic film lubrication. J. Mol. Liq. **128**, 60–64 (2006)
13. Zhang, Y.B.: The Reynolds equation for boundary film considering the non-continuum effect and its application to the one-dimensional micro step bearing: part ii-calculation for boundary slippage. J. Comput. Theor. Nanosci. **10**, 609–615 (2013)
